# The complete mitochondrial genome of *Gymnogobius heptacanthus* (Perciformes, gobiidae)

**DOI:** 10.1080/23802359.2016.1247671

**Published:** 2016-11-11

**Authors:** Ha Yeun Song, Young Se Hyun, Moongeun Yoon, Jiyoung Woo, Byung-Jin Lim, Hyung June Kim, Hye Suck An

**Affiliations:** a Genetic Resources Team, National Marine Biodiversity Institute of Korea, Seocheon-gun, Republic of Korea;; b Marine Biodiversity Research Division, National Marine Biodiversity Institute of Korea, Seocheon-gun, Republic of Korea;; c Marine Invertebrate II Team, National Marine Biodiversity Institute of Korea, Seocheon-gun, Republic of Korea;; d Marine Invertebrate I Team, National Marine Biodiversity Institute of Korea, Seocheon-gun, Republic of Korea

**Keywords:** Mitochondrial genome, perciformes, gobiidae, *Gymnogobius heptacanthus*

## Abstract

*Gymnogobius heptacanthus* is a small intertidal species belonging to the family Gobiidae. Herein, we report the first sequencing and assembly of the complete mitochondrial genome of *G. heptacanthus*. The complete mitochondrial genome is 16,529 bp long and has the typical vertebrate mitochondrial gene arrangement, consisting of 13 protein-coding genes, 22 tRNA genes, 2 rRNA genes, and a control region. Phylogenetic analysis using mitochondrial genomes of 12 species showed that *G. heptacanthus* is clustered with *G. urotaenia* and *G. petschiliensis* and rooted with other Gobiidae species. This mitochondrial genome provides potentially important resources for addressing taxonomic issues and studying molecular evolution.

*Gymnogobius heptacanthus* is a small intertidal species belonging to the family Gobiidae. It is widely distributed in the salt and brackish waters of Korea, Japan, and China, and is one of the main contributors to the biodiversity in these habitats (Dotsu 1984). However, due to the difficulties of species identification using morphological characters (Thacker & Roje 2011), the phylogenetic position of *G. heptacanthus* within the Gobiidae remains unclear. To the best of my knowledge, this is the first study that has sought to determine the complete mitochondrial genome of *G. heptacanthus*, and to analyze the phylogenetic relationship of this species among Gobiiformes fishes.

The *G. heptacanthus* specimen used in this study was collected from Gyeokpo-ri, Buan-gun, Jeollabuk-do, South Korea (35.37N, 126.28E). Total genomic DNA was extracted from tissues of the specimen, which has been deposited in the National Marine Biodiversity Institute of Korea (Voucher No. MABIK TV00000002). The mitogenome was amplified in its entirety using a long PCR technique (Cheng et al. 1994). We used fish-versatile PCR primers in various combinations to amplify contiguous, overlapping segments of the entire mitogenome. All the experiments were carried out following previously described methods (Miya & Nishida 1999).

The complete mitochondrial genome of *G. heptacanthus* (GenBank accession no. AP017651) is 16,529 bp long, and includes 13 protein-coding genes, 22 tRNA genes, 2 rRNA genes, and a control region. The *ND6* gene and eight tRNA genes are encoded on the light strand. The overall base composition of the heavy strand is 27.23% A, 27.31% C, 16.84% G, and 28.61% T. Similar to the mitogenomes of other vertebrates, the AT content is higher than the GC content (Saccone et al. 1999). All tRNA genes can fold into a typical cloverleaf structure, with lengths ranging from 65 to 75 bp. The 12S rRNA (950 bp) and 16S rRNA genes (1683 bp) are located between tRNA^Phe^ and tRNA^Val^ and between rRNA^Val^ and tRNA^Leu(UUR)^, respectively. Of the 13 protein-coding genes, 12 begin with an ATG start codon; the exception being the *COI* gene, which starts with GTG. The stop codon of the protein-coding genes is TAA in *NDI*, *COI*, *ATP8*, *ATP6*, *ND4L*, and *ND5*; TAG in *ND6*; TA in *ND2*; and T in the remaining five genes. The control region (878 bp) is located between tRNA^Pro^ and tRNA^Phe^.

Phylogenetic trees were constructed by the maximum-likelihood method with 1000 replicates using MEGA 7.0 software (MEGA, PA, USA) (Kumar et al. 2016) for the newly sequenced genome and a further 11 complete mitochondrial genome sequences downloaded from the National Center for Biotechnology Information. We confirmed that *G. heptacanthus* is clustered with *G. urotaenia* and *G. petschiliensis* (Kim et al. 2004) and rooted with other Gobiidae species (Figure 1). This mitochondrial genome provides potentially important resources for addressing taxonomic issues and studying molecular evolution.

**Figure 1.**
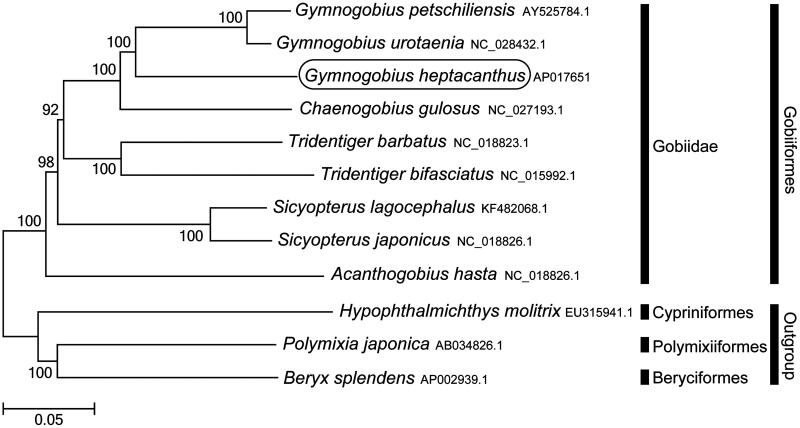
Phylogenetic position of *Gymnogobius heptacanthus* based on a comparison with the complete mitochondrial genome sequences of 11 species. The analysis was performed using MEGA 7.0 software. The accession number for each species is indicated after the scientific name.
